# Analytical chemistry with biosolvents

**DOI:** 10.1007/s00216-019-01732-2

**Published:** 2019-03-26

**Authors:** Marek Tobiszewski

**Affiliations:** Department of Analytical Chemistry, Chemical Faculty, Gdańsk University of Technology (GUT), 11/12 G. Narutowicza St., 80-233 Gdańsk, Poland

**Keywords:** Biobased solvents, Biobased economy, Green analytical chemistry, Renewable resources, Biorefinery, Extraction

## Abstract

One of the current trends in green analytical chemistry is the introduction of green solvents, some of which are biobased. At the same time, the development of the biorefinery concept has allowed more biochemicals to be obtained with increased efficiency and from a wider range of feedstocks. The first examples of the use of biosolvents in analytical applications included extractions performed with alcohols, esters, and terpenes. However, many more applications of biosolvents in extractions of bioactive compounds from various plant materials have also been reported, which hints at a wider range of potential analytical applications of biosolvents. It should also be noted that the biobased solvents applied in analytical chemistry are not always green, as some of them are toxic towards aquatic organisms.

**Figure:**
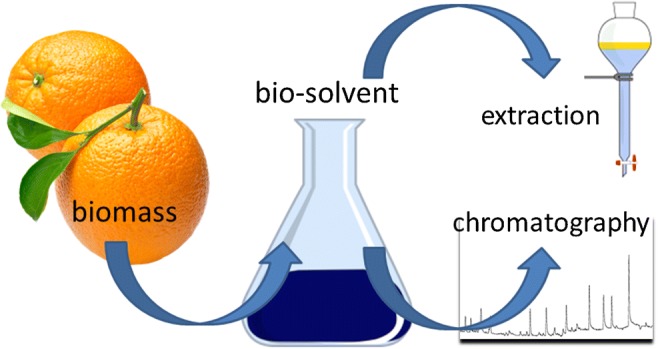
Graphical abstract

Graphical abstract

## Introduction

The concept of green analytical chemistry (GAC) involves the introduction of the 12 principles of green chemistry into analytical laboratories [1]. The main aims of this push for greener chemistry are the miniaturization of analytical techniques, especially extraction techniques, the simplification of procedures in order to avoid unnecessary steps, and the application of less hazardous reagents, derivatization agents [2], and solvents [3]. The optimal way to implement the principles of GAC is to use solventless extraction techniques. It should be noted that the concept of cleaner analytical chemistry is not new; it was intuitively introduced into analytical chemistry long before the acronym “GAC” was first used.

One of the trends in modern analytical chemistry is the introduction of green solvents [4]. These can be less harmful organic solvents, ionic liquids, deep eutectic solvents (DESs), or supercritical fluids [5]. However, the latter three examples require the application of complex devices or modified equipment, or their greenness has been questioned, as in the case of ionic liquids, given that they are generally characterized by high toxicity towards aqueous organisms [6]. While the negligible vapor pressures of ionic liquids are a favorable characteristic, because there is then no possibility of exposure through inhalation, this property makes ionic liquids incompatible with gas chromatography. DESs are a class of ILs that are usually relatively cheap and easy to produce, and are biodegradable [7]. Another advantage is the possibility of obtaining DESs from biosources; this is particularly true of choline derivatives, organic acids, and aminoacids [8]. The main limitations on their utilization in analytical chemistry are their high viscosities and their solid state at room temperature. Among supercritical fluids, the most commonly used compound is carbon dioxide, which is nontoxic, incombustible, easily available, and cheap. Its properties can be altered by changing the temperature and pressure, and it can easily be separated from the extract by relaxing the conditions to room temperature and pressure [9]. The main limitations of supercritical fluid technology are the need for sophisticated equipment and its high energy demands. We can therefore conclude that the main focus of the search for practicable green solvents should be less harmful organic solvents.

One of the main lines of green solvent development is the application of biobased solvents that are of renewable origin (which are usually readily biodegradable and less toxic than most of the solvents that derive from petroleum-related sources) in both analytical chemistry [8] and general green chemistry [10]. As the practical application of biobased solvents appears to be on the near horizon in many areas of chemical technology, the potential applications of these solvents in analytical chemistry have been largely overlooked. The main potential applications of biobased reagents in analytical chemistry relate to spectrophotometric and electrochemical techniques. Indeed, as discussed in [11], natural products can be used directly or with minor preparation as reagents in many analytical procedures, but the main issue encountered when applying them is a tendency for higher limits of detection.

One of the current trends in chemical technology is to shift the sources of chemical feedstocks from fossil fuels to biobased ones [12]. Biomass, a by-product of many areas of agriculture and industry, is valorized to obtain useful chemicals or fuels. Industrial biomass valorization is currently undergoing rapid development; a wider range of biomass feedstocks are being considered and a greater fraction of the biomass is being valorized. The shift from petroleum-based sources towards renewable sources of materials also seems to be unavoidable for analytical laboratories. The crucial question to answer in this regard is if analytical chemistry no longer needs to rely on petroleum-based solvents. In other words, the question is whether analytical chemistry can manage in a biobased economy, and even benefit from it.

## Feedstocks and a brief introduction to the biorefinery concept

Most of the chemicals currently produced industrially are petrogenic in origin. The transition from a fossil-fuel-based economy towards a biobased economy is unavoidable in the case of chemical and fuel production. To meet these demands, the concept of biorefineries has been intensively developed in recent years. The products of the processing of different feedstocks by biorefineries can be of energetic value or of material value. The feedstock—the raw material that is processed in a biorefinery, analogously to how crude oil is refined in a traditional refinery—can be obtained from various sources. The most common of these are [13]: agricultural, such as waste from production (which frequently originates from citrus processing [14]) or from crops grown for this purpose; forestry and wood processing; processing waste from other industries, and biomass of marine origin, such as algae.

The three main pathways to obtaining solvents from biosources are the fermentation of carbohydrates, vegetable oils, and the steam distillation of wood [15]. In the second case, the products of biomass processing are platform chemicals that are subsequently used in syntheses of desirable products. Examples of building block chemicals used in biosolvent production from lignocellulosic mass include the following [16]:

- 3-Hydroxypropionic acid, with 1,3-propanediol, propiolactone, and malonic acid as end products
- *n*-Butanol, ethanol, methanol, and *n*-propanol, with the same or other molecules (such as acetic acid, acetone, acetaldehyde, ethyl acetate, butyl acetate, isobutene, or *p*-xylene) as final products
- Furans, with end products such as formic acid and tetrahydrofuran
- Lactic acid, with butyl lactate, ethyl lactate, and propyl lactate as end products
- Succinic acid, with end products of 1,4-butanediol and tetrahydrofuran

Another important product that is widely recognized to be a chemical derived from biomass is ethanol. It is obtained from sugar-containing plants or plant waste, lignocellulosic mass, marine algae and microalgae, straw, and other sources. Solvents that can be obtained from bioethanol include ethyl esters (through ethylene), acetic acid, *n*-butyl acetate (through ethanal), and other esters (through acetic acid) [17].

Another biobased solvent worth mentioning, ethyl lactate, is obtained from corn and soybeans by fermenting the biomass and reacting two fermentation products—ethanol and lactic acid [18]. As it is biodegradable, nontoxic, and exhibits relatively low volatility, ethyl lactate is used to extract phytochemicals [19].

## Biosolvents used in analytical chemistry

### Extraction with biobased solvents

There are already analytical procedures in which traditional solvents have been replaced with their biobased counterparts. One example of a biobased solvent already used in analytical practice is D-limonene, which is employed instead of toluene to determine moisture with the Dean–Stark apparatus [20]. Importantly, this substitution was recommended by the American Oil Chemists’ Society (AOCS) for official method Ja 2a-46, which is used routinely. Changing solvent did not significantly affect procedural performance. This terpene has also been applied instead of *n*-hexane for the determination of fats and oils in olive seeds [21]. Apart from switching to a less toxic and less hazardous solvent, the proposed procedure included a successful solvent recycling step. Thus, the possibility of recovering and reusing  D-limonene is another advantage. It has also been used as substitute for *n*-hexane when determining the total lipids in various foods [22]. The environmental impact of the extraction solvent is reduced by selecting the (relatively) benign D-limonene as solvent as well as by significantly reducing the volume of solvent used. This can be achieved through the use of microextraction techniques, such as dispersive liquid–liquid microextraction (DLLME) [23]. β-Cyclodextrin was determined using a DLLME method in which 200 μL of D-limonene were employed as extraction solvent and 1 mL of acetone as dispersive solvent. DLLME combined with spectrophotometric determination yielded an acceptable LOD and linear range. Interestingly, β-cyclodextrin forms a colored complex with β-carotene, a bioreagent obtained from carrots, which means that the analytical procedure is completely biobased. However, it should be mentioned at this point that D-limonene is highly toxic towards aquatic organisms [24].

Menthol has also been used as a biobased extraction solvent in the DLLME of five phthalate esters [25]. Fifty milligrams of menthol were applied together with 1.25 mL of acetone, and the resulting LODs were within the range 1–8 μg L^−1^. The precision, expressed as the coefficient of variance, was within the range 4–8%. Diethyl carbonate, a green solvent that can be synthesized from ethanol, has been applied to extract chlorophenols from water using DLLME [26]. Metrological values were comparable with those obtained using other analytical procedures based on extraction with toluene, chlorobenzene, or butyl acetate.

A polyethylene glycol water solution was applied for the microwave-assisted extraction of flavones and coumarin from plant samples [27]. This procedure permitted almost 100% efficient extraction in 2–6 min depending on the analyte, and the performance was better than that achieved with methanol.

Several biosolvents were tested for the extraction of biocides from fish tissue samples via vortex-assisted matrix solid-phase dispersion extraction [28]. Almost all of the investigated solvents—acetone, hexane, ethyl acetate, tetrahydrofuran, a methanol–acetic acid mixture, ethanol, methanol (note that all of these are biosolvents except for hexane)—permitted extraction efficiencies of >96%. As these solvents all met the required analytical performance criteria, other factors were considered when selecting the extraction solvent. Ultimately, ethanol was chosen, as it was the least toxic and has beneficial physicochemical properties. Another study showed that ethyl acetate is the best option for extracting fatty acids from salmon tissues [29], as it was found to allow a slightly better extraction efficiency than those attained with 2-methyltetrahydrofuran, cyclopentyl methyl ether, dimethyl carbonate, isopropanol, ethanol, ethyl acetate, *p*-cymene, and D-limonene.

An investigation aiming to discern the best biosolvent from among ethanol, D-limonene, and ethyl lactate to apply in the pressurized liquid extraction of thymol from thyme essential oil [30] showed that all three biosolvents facilitated the extraction of thymol, but D-limonene was observed to give the best response, due to its lipophilic properties.

It should be noted that the number of analytical procedures that utilize biosolvents is still not very high. Biosolvents are more widely applied in other branches of chemistry, especially to obtain biologically active compounds from plant materials. This fact demonstrates the potentially wide applicability of biosolvents in analytical extractions, albeit under two conditions. The first is that the extraction efficiency of target compounds should be close to 100%. The second condition is that it is possible to obtain a high-purity biosolvent or a biosolvent contaminated with compounds that are not interferents. When the biosolvent is used in chromatographic techniques, any contaminants of the biosolvent must not elute together with the analyte, and when the final determination is made spectrophotometrically, the contaminants must not absorb radiation at the wavelength at which the analyte absorbs. Biosolvent purification can be achieved through well-established methods such as distillation or the application of a dehydrating or deoxygenating agent [31].

An example of a biosolvent applied in plant material processing is eucalyptol, which can be as effective at extracting phenolic compounds from algae as a 1:1 v/v mixture of dichloromethane–methanol mixture [32]. Another example is the application of methyltetrahydrofuran for the extraction of steroids from rapeseeds as efficiently as *n*-hexane [33]. A chloroform–methanol mixture or *n*-hexane can be replaced with 2-methyltetrahydrofuran–isoamyl alcohol or 2-methyltetrahydrofuran–ethanol [34], or with ethanol and ethyl acetate [35], for the extraction of lipids from microalgae.

### Biobased versus green solvents

The solvents that are generally applied as mobile phases in liquid chromatography are methanol, ethanol, and acetonitrile. The latter cannot be considered a biosolvent, and it causes more environmental problems than the other two solvents. The application of methanol and ethanol with appropriate modifiers should give satisfactory results in the majority of liquid-chromatographic separations. There are also plenty of polar solvents that are applied to extract polar or moderately polar compounds from many solid matrices. It may be more problematic to find suitable nonpolar biobased solvents for the extraction of nonpolar analytes from water samples. The main options in this area are terpenes, which are associated with certain environmental issues [36]. The main concern here is their high potential to facilitate the generation of photochemical smog, which reminds us that a solvent derived from a biosource is not necessarily green. This issue has already been discussed in relation to furfural, which is obtained from biosources but is considered to be toxic and carcinogenic [37]. Many of the solvents included in Table 1 have been investigated regarding their applicability as green solvents [38], and the findings support the statement that analytical chemistry lacks opportunities for the application of nonpolar biobased solvents except for terpenes. Table 1 lists biosolvents together with their greenness scores, their toxicities towards fish, and their biodegradabilities. While most of the biobased solvents are readily biodegradable, some of them (such as terpenes) are toxic towards fish. Results for the GSK assessment of solvent greenness indicate that some of the biobased solvents are problematic.

**Table 1:** Basic greenness parameters of biobased solvents

Biosolvent	Greenness assessment according to [24]	Toxicity towards fish (LC_50_, in mg L^−1^)	Biodegradability half-life (days)
Acetaldehyde	Not included	31	7
Acetic acid	Problematic	1000	2
Acetone	Recommended	5500	3
*i*-Butanol	Recommended	1120	7
*n*-Butanol	Recommended	1200	7
*n*-Butyl lactate	Not included	75	10
Ethanol	Recommended	14,500	2
Ethyl acetate	Recommended	350	5
Ethyl lactate	Problematic	320	14
D-Limonene	Problematic	0.7	7
α-Pinene	Not included	0.28	21
*n*-Propanol	Not included	4500	3
Tetrahydrofuran	Problematic	2160	40

Some authors state that the greenness comparison should be contextualized and that the assessment should be done for a particular process or application (see the case study of ILs in [39]). The assessment of biobased solvents is a multistep process in which the number of candidates is trimmed at each step and the candidates are considered in increasing detail as the process proceeds [40]. Most of the steps are relevant to analytical applications: selection of replacement candidates, in silico prediction of properties, testing physical properties, toxicology assessment, and greenness and life-cycle assessment.

The benefits of deriving solvents from carbohydrates in corn, from lignin in biomass, and from carbohydrate non-corn feedstocks were assessed based on a comparison of life-cycle greenhouse gas emissions with those of their fossil fuel-based counterparts (expressed in percent, with positive values showing increased greenhouse gas emissions and negative values indicating decreased emissions) [41]. Acetic acid obtained from corn carbohydrate is associated with a 44% increase in emissions, whereas the values for ethyl lactate and *n*-butanol are −87 and − 66%, respectively. Obtaining methanol from lignin-based material results in a 76% decrease in greenhouse gas emissions. The values for solvents obtained from non-corn carbohydrate-rich materials (sugarbeet, potato juice, potato molasses, sugarcane, and switchgrass) were −168% for *n*-butanol, −94% for *i*-butanol, −7% for acetic acid, and 12% for *p*-xylene. This shows that biobased solvents are not always greener options, or are at least not greener in all aspects.

The use of D-limonene derived from citrus waste as a substitute for toluene in cleaning applications shows that biosolvent utilization is feasible only in areas that generate high levels of citrus waste [42].

## Outlook

Based on the aspects described above, we can expect the use of biobased organic solvents in analytical applications to gain in popularity. However, the driving force for the development and application of new biosolvents is (green) chemical technology, not analytical chemistry. Therefore, analysts who wish to apply new biobased solvents or develop novel analytical procedures based on such solvents need to monitor trends in green chemistry and technological separation processes. Greenness assessments of biosolvents should be supported by fast computational methods, such as quantitative structure–activity relationships [43]. The search for water-insoluble and green biosolvents that are applicable to extractions is particularly important, as such solvents are urgently needed.

The development of and research into applications of biosolvents in analytical extractions and related fields are becoming increasingly noticeable. The focus so far has largely been on alcohols, esters, and terpenes. It appears that some of the solvent requirements of analytical chemistry can be satisfied by polar biosolvents, which are applied as extraction solvents or mobile-phase constituents in liquid chromatography, but it is difficult to find nonpolar biosolvents that are applicable in extractions of lipophilic substances from polar media. The present discussion of biosolvents shows that most of them (especially the polar ones) can be treated as greener solvents, but also that biobased solvents are not automatically green (this is true of terpenes, for instance).
